# Associations between ATM c.7271T>G and cancer risk: analysis of Breast Cancer Association Consortium and UK Biobank data

**DOI:** 10.1136/jmg-2025-110769

**Published:** 2025-06-01

**Authors:** Toqir K Mukhtar, Leila Dorling, Naomi Wilcox, Joe Dennis, Xin Yang, Melissa Southey, Marc Tischkowitz, Douglas F Easton

**Affiliations:** 1 Centre for Cancer Genetic Epidemiology, Department of Public Health and Primary Care, Strangeways Research Laboratory, University of Cambridge, Cambridge, UK; 2 Department of Primary Care and Public Health, Imperial College London, London, UK; 3 Precision Medicine, School of Clinical Sciences at Monash Health, Monash University, Clayton, Victoria, Australia; 4 Cancer Epidemiology Division, Cancer Council Victoria, Melbourne, Victoria, Australia; 5 Melbourne School of Population and Global Health, The University of Melbourne, Parkville, Victoria, Australia; 6 Genomic Medicine, National Institute for Health Research Cambridge Biomedical Research Centre, University of Cambridge, Cambridge, UK; 7 Centre for Cancer Genetic Epidemiology, Department of Oncology, University of Cambridge, Cambridge, UK

**Keywords:** Human Genetics

## Abstract

Previous studies have suggested the missense variant NM_000051.4(ATM):c.7271T>G is associated with a high risk of breast cancer (BC), but the magnitude of the association, and the associations with other cancer types, are unclear. Cancer associations were evaluated using sequence data linked to cancer registration data (348 488 participants, 56 640 cancer cases) from UK Biobank (UKB), and targeted sequence or genome-wide array data (126 428 cases, 115 495 controls) from the Breast Cancer Association Consortium (BCAC). The magnitudes of the association of c.7271T>G with invasive BC were similar using UKB (relative risk (RR): 4.57, 95% CI: 2.25 to 9.30, p=2.7×10^−5^) and BCAC (OR: 4.11, 2.05 to 8.26, p=6.9×10^−5^). In UKB, c.7271T>G was associated with increased risks of prostate cancer (4.84, 2.27 to 10.33, p=4.54×10^−5^), and any other cancer (males 2.79, 1.33 to 5.85, p=0.0066; females 3.15, 1.49 to 6.63, p=0.0026). Estimated cumulative risks of all cancers to age 80 years were 87% in males (prostate cancer 43%) and 84% in females (BC 43%). The estimated RRs are consistent with c.7271T>G being associated with a risk of more than twice that for Ataxia–Telangiectasia Mutated protein-truncating variants, for all cancers. These data justify specific management of c.7271T>G carriers.

## Introduction

 Biallelic germline pathogenic variants in *ATM* (Ataxia-Telangiectasia Mutated) cause A-T (Ataxia-Telangiectasia), a rare autosomal recessive neurodegenerative disorder associated with immunodeficiency and high cancer risk.^1^ Heterozygous carriers of pathogenic variants (principally protein truncating variants, PTVs) in *ATM* have been shown in multiple studies to be associated with a moderately increased risk of breast cancer (BC), with a relative risk (RR) of approximately 2.^2 3^ Recent studies have also shown clear associations between *ATM* PTVs and a wide range of other cancer types, notably prostate,^4 5^ gastric, pancreatic,^5 6^ colorectal,^7^ lung, bladder, ovarian and oesophageal cancer, diffuse non-Hodgkin’s lymphoma, lymphoid leukaemia, and melanoma.^5^

There is also evidence that a subset of *ATM* missense variants is associated with risk of multiple cancers including breast, prostate, and pancreatic cancer.^8 9^ The risk for BC appears to be restricted to a subset of missense variants lying in the FRAP-ATM-TRRAP (FAT) and phosphatidylinositol 3-kinase (PI3K) domains of *ATM*; for these variants, the risk appears to be comparable with the risks for PTVs.^5 9^

One *ATM* missense variant that has been the focus of much research is NM_000051.4(ATM):c.7271T>G (p.Val2424Gly) (henceforth called c.7271T>G) which lies in the FAT domain. This variant was originally identified through studying a family with A-T, in which homozygous or compound heterozygous variant carriers had a milder A-T phenotype.^10^ Evidence from this and subsequent family studies has suggested that this variant is associated with a high risk of BC.^10 11^ Some studies have reported risks of BC for carriers with odds ratios (ORs) >10.^11 12^ However, the risk estimates are from relatively small studies, or studies of high-risk families where cases are oversampled compared to the population, potentially resulting in an upward bias of the risk ratio.

Aside from the association with BC risk, evidence is much more limited for other possible cancer associations. There is some evidence to suggest that the c.7271T>G variant is associated with increased risk of pancreatic, prostate, and stomach cancer (with ORs>2),^13^ but the risks of all cancers have not been examined systematically.

In this paper, we use data from two large resources, Breast Cancer Association Consortium (BCAC) and UK Biobank (UKB), neither of which oversampled for high-risk families, to provide more reliable estimates for the relative and absolute risks of BC and for other cancer types associated with c.7271T>G.

## Materials and methods

### Data sources

#### Breast Cancer Association Consortium

We analysed data for female participants from three projects that provided genotype data for c.7271T>G (online supplemental table 1): the BRIDGES project generated data using a targeted sequencing panel of BC susceptibility genes for 37 999 population-based invasive BC cases, 2047 breast carcinoma in-situ (CIS) cases, and 42 941 controls from 37 studies^3^; the OncoArray project generated array genotyping data for 53 489 invasive BC cases, 5194 CIS cases, and 47 214 controls from 76 studies^14^; and the Collaborative Oncological Gene-environment Study (COGS) generated genotypes for 23 295 invasive BC cases, 4404 CIS cases, and 25 340 controls from 43 studies.^15^ Details regarding data collection, genotyping, and quality control procedures for each study have been published previously.^3 14 15^ The large majority of studies in the OncoArray project were population-based case-control studies. Individuals included in more than one project were preferentially included in the BRIDGES and then the OncoArray datasets. Since no c.7271T>G variant carriers were identified in individuals of non-European ancestry, analyses were restricted to individuals of European ancestry, determined by genotyping where available, or self-reported ethnicity. Both the OncoArray and iCOGS array include the c.7271T>G variant in the design. Cluster plots indicate that the variant was well genotyped (online supplemental figure 1a and 1b), and genotypes for samples which overlapped between the array genotyping and the BRIDGES targeted sequencing were concordant.

#### UK Biobank

UKB is a prospective, population-based study of approximately 500 000 participants recruited in the UK, with whole-exome sequencing data on 454 756 participants.^16^ UKB allows for both a retrospective and prospective component. Retrospective analyses were based on cases diagnosed with cancer and unaffected controls before entry to the study, and included 348 488 individuals, with 18 838 diagnosed with at least one cancer before the day of first assessment. Prospective analyses were based on participants unaffected with cancer (except non-melanoma skin cancer) at study entry or in the first 6 months after entry, and included 328 919 individuals. Follow-up was continued to the first diagnosed cancer of any type, date of death, or a last follow-up date as previously described.^5^ A total of 37 802 cancers were diagnosed over 3 484 613 person years of follow-up. Information on study design and variant filtering has been published previously.^5^

## Statistical analyses

### Breast Cancer Association Consortium

The primary analysis was performed on the three BCAC datasets combined. Case-control analyses were performed using logistic regression, with invasive BC or CIS as the outcome and c.7271T>G carrier status as a covariate. All analyses were adjusted for country and genotyping platform. In sensitivity analyses, models were also adjusted for principal components (PCs) for OncoArray (10 PCs) and iCOGS (9 PCs).^14 15^ The trend in the OR associated with c.7271T>G by age was evaluated in the combined dataset using a case-only analysis. Case-control analyses were also performed by oestrogen receptor (ER) status. There were too few data to examine more detailed subtypes.

### UK Biobank

For the retrospective analyses, logistic regression was used to estimate the OR between carrying the c.7271T>G variant and any cancer type. In the prospective dataset, Cox proportional hazards regression was used to estimate the HR associated with carrying the c.7271T>G and any cancer type. Models were adjusted for 10 PCs and sex. Overall RR estimates were obtained by combining the OR from the retrospective analyses and the HR from the prospective analyses using a fixed-effect meta-analysis. For BC, this estimate was also combined with the BCAC OR estimate.

Cumulative risks were estimated with the HRs from the prospective analyses from UKB combined with the ORs from BCAC for BC, and cancer incidences in the UK,^17^ as previously described.^5^ To estimate cumulative risks for BC allowing for an age-dependent RR, the ORs and HRs by age were re-estimated fixing the trend in the log(OR) by age estimated from the case-only analysis in BCAC.

All analyses were conducted using R version 4.1.0. Meta-analyses were performed using the meta package version 6.5-0, and the metagen function. All p values were two-sided, with p<0.05 considered significant.

## Results

### Breast cancer

In total, 60 carriers of c.7271T>G were identified in the BCAC dataset (43 invasive BC cases, 7 CIS cases, and 10 controls). All but three carriers were from the UK, the USA, Australia, or Canada. In the BCAC dataset (table 1), c.7271T>G was associated with an increased risk of invasive BC (OR: 4.11 (95% CI 2.05 to 8.26, p=6.9×10^−5^)). There was no evidence of heterogeneity in the OR among the three projects: in particular, the estimate from BRIDGES, though lower, is compatible with the combined estimate (I^2^=36.11, p=0.21, online supplemental table 2). A sensitivity analysis in which the combined analysis was adjusted for PCs gave similar results (online supplemental table 2). In a case-only analysis, the OR for invasive BC decreased with increasing age (per year, OR: 0.95 (0.92 to 0.97, p=4.9×10^−5^)).

**Table 1:** Estimated pooled (ORs) and relative risks (RRs) for the association of c.7271T>G with invasive breast cancer (BC) and carcinoma in-situ of the breast (CIS), in the BCAC and UKB datasets

Outcome	Dataset	Case carriers	Case non-carriers	Control carriers	Control non-carriers	OR/RR	95% CI	P value
Invasive BC	BCAC	43	114 736	10	115 482	4.11	2.05 to 8.26	6.9×10^−5^
	UKB	8	12 495			4.57	2.25 to 9.30	2.7×10^−5^
	Combined	51	127 231			4.37	2.65 to 7.20	6.9×10^−9^
ER+BC	BCAC	34	80 196	10	115 482	8.34	3.22 to 21.6	1.2×10^−5^
ER−BC	BCAC	2	20 102	10	115 482	2.26	0.43 to 11.9	0.34
CIS	BCAC	7	11 638	10	115 482	9.30	2.69 to 32.2	4.3×10^−4^
CIS	UKB	3	2063			11.1	3.55 to 34.8	3.6×10^−5^
CIS	Combined	10	13 701			10.3	4.44 to 23.7	4.9×10^−8^

BCAC, Breast Cancer Association Consortium; UKB, UK Biobank.

In UKB, 65 carriers were identified in the retrospective dataset and 58 in the prospective dataset. The c.7271T>G variant was associated with BC in both datasets (combined RR: 4.57 (2.25 to 9.30, p=2.7×10^−5^)) (online supplemental table 3). There was no significant difference between the OR in the retrospective component and the HR in the prospective component, nor between the BCAC and UKB estimates (combined RR: 4.37 (2.65 to 7.20, p=6.9×10^−9^)).

There was evidence for an association between c.7271T>G and CIS in both the BCAC (OR: 9.30 (2.69 to 32.2, p=4.3×10^−4^)) and UKB datasets (HR: 11.12 (3.55 to 34.84, p=3.6×10^−5^); combined RR: 10.25 (4.44 to 23.7, p=4.9×10^−8^)). In the BCAC subtype analysis, c.7271T>G was associated with increased risk of ER-positive (OR: 8.34 (3.22 to 21.6, p=1.2×10^−5^)) but not ER-negative disease (table 1).

In the pooled analyses in UKB (online supplemental table 3), c.7271T>G was associated with prostate cancer (RR: 4.84 (2.27 to 10.3, p=4.5×10^−5^)). There was some evidence of an excess of cancers other than breast and prostate cancer (combined RR females: 3.15 (1.49 to 6.63, p=0.0026) and males: 2.79 (1.33 to 5.85, p=0.0066)). Eleven cancers, other than breast and prostate cancer, occurred in carriers (online supplemental table 4). Of note, two of these were pancreatic cancer.

**Figure 1:**
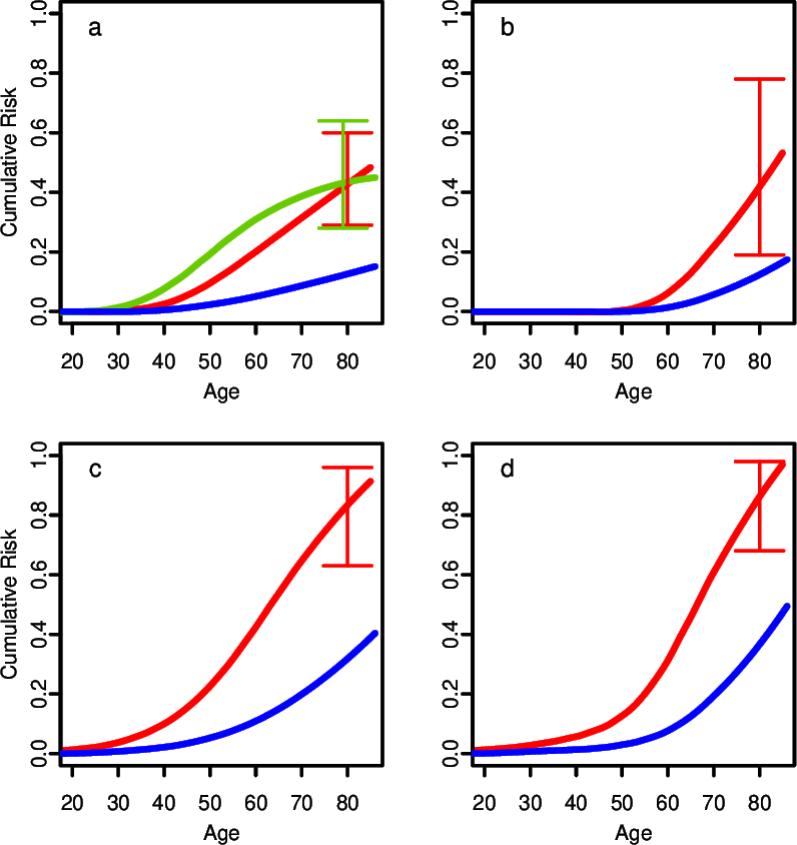
Cumulative risks of (a) breast cancer (BC) in females, (b) prostate cancer, (c) all cancers in females, and (d) all cancers in males. Red lines are cumulative risks for c.7271T>G carriers under a constant relative risk (RR) assumption. Green line for BC is the cumulative risk under an age-dependent RR assumption. Blue lines are the corresponding population risks (see Materials and methods section). Error bars are 95% confidence limits.

Based on the combined BCAC/UKB RR estimate, the cumulative risk of BC in c.7271T>G carriers by age 80 was estimated to be 43% (95% CI: 29% to 60%) compared to 12% in the general population of the UK (figure 1a). Allowing for the trend by age estimated in the BCAC dataset, the cumulative risk would be 44% (28% to 64%) by age 80. For prostate cancer, the cumulative risk by age 80 was 43% (19% to 78%), compared to 14% in the general population (figure 1b). The cumulative risk of any cancer by age 80 was 84% (63% to 96%) in females and 87% (68% to 98%) in males, compared to 33% for females and 39% for males in the general population (figures 1c and 1d, respectively).

## Discussion

Both the BCAC and UKB datasets provide strong evidence for an association between c.7271T>G and BC. The overall RR estimates were similar, with a combined RR of 4.37 (2.65 to 7.20, p=6.9×10^−9^). This estimated risk was more than twice that typically estimated for *ATM* PTVs, for example, from BRIDGES Study (OR: 2.10, 1.71 to 2.57).^3^ These results provide strong confirmation that this variant is associated with a higher risk of BC than PTVs. However, the upper 95% confidence limit would exclude a 10-fold risk suggested in some previous analyses. There was also strong evidence of an association with ER positive, but not ER negative, BC, and CIS, though the CIs were wide. Similarly, there was good evidence for an association of c.7271T>G with prostate cancer (RR=4.8 vs 2.4 for PTVs in a previously published study).^5^

In addition to breast and prostate cancer, the UKB analyses show a clear excess of other cancers with risks (approximately threefold) also higher than for *ATM* PTVs.^5^ It is not possible to provide reliable estimates for individual cancers, but 9 of the 11 cancers were at sites that are also associated with *ATM* PTVs, including stomach, pancreas, lymphomas, and leukaemia. Taken together, these observations are broadly consistent with the hypothesis that c.7271T>G is associated with the same range of cancers as PTVs, but is associated with more than twice the risk of all cancer types. The mechanisms underlying this higher risk are unclear, but previous analysis in lymphoblastoid cell lines has suggested that this variant can act in a dominant-negative manner, so that DNA damage response may be abrogated without inactivation of the wild-type allele.^11^

Confidence limits are still wide and further large prospective studies will be needed to provide more precise estimates for individual cancers and cancer subtypes, and to determine the extent to which these risks are modified by other factors. Further large prospective studies, including populations of non-European ancestry, may identify other variants of similar risk.

The absolute BC risk (~44% by age 80) would classify c.7271T>G a ‘high-risk’ variant according to National Institute for Health and Care Excellence (NICE) guidelines.^18^ This risk estimate is comparable to that associated with deleterious variants in *PALB2*, though lower than the corresponding estimates for *BRCA1* and *BRCA2*. The prostate cancer risk (43% by age 80) is also strikingly high and comparable to that for *BRCA2* PTV carriers.^19^ Recently published international guidelines on the clinical management of *ATM* heterozygotes indicated that additional cancer surveillance and prevention options may be considered for those with higher risk *ATM* variants, including c.7271T>G.^20^ Given the very high overall risk of cancer in both males and females seen here, our results add further weight to those recommendations.

## Supplementary material

10.1136/jmg-2025-110769 online supplemental file 1
