# Supplementary material for: Associations between ATM c.7271T>G and cancer risk: analysis of Breast Cancer Association Consortium and UK Biobank data
Source: J Med Genet. 2025 Jun 1;62(9):e110769. doi: 10.1136/jmg-2025-110769 (PMC12418526; doi:10.1136/jmg-2025-110769)

Associations between *ATM* c.7271T>G and cancer risk: analysis of Breast Cancer Association Consortium and UK Biobank data

Toqir K. Mukhtar, Leila Dorling, Naomi Wilcox, Joe Dennis, Xin Yang, Melissa Southey, Marc Tischkowitz, Douglas F. Easton

Supplemental tables

Supplemental table 1: BCAC participating studies with numbers of cases and controls

Supplemental table 2: Estimated odds ratios for the association of *ATM* c.7271T>G with invasive breast cancer (BC) and carcinoma in-situ of the breast (CIS), by panel, in the BCAC dataset

Supplemental table 3: Odds ratios, hazard ratio, and pooled estimates for the association of c.7271T>G in *ATM* with cancer by type in UKB whole-exome sequence data

Supplemental table 4: Other cancers identified in *ATM* c.7271T>G carriers in UKB data

Supplemental table 1: BCAC participating studies with numbers of cases and controls [1]

Study[1]	Country	Panel	Cases[2]	Controls
ABCS	Netherlands	BRIDGES, OncoArray, iCOGS	1149	1821
BBCC	Germany	BRIDGES, OncoArray, iCOGS	947	712
BIGGS	Ireland	BRIDGES, iCOGS	839	722
BREOGAN	Spain	BRIDGES, OncoArray	1667	917
BSUCH	Germany	BRIDGES, OncoArray, iCOGS	1152	1147
CCGP	Greece	BRIDGES, OncoArray	685	353
CECILE	France	BRIDGES, OncoArray, iCOGS	1059	1035
CGPS	Denmark	BRIDGES, OncoArray, iCOGS	4361	10326
CNIO-BCS	Spain	BRIDGES, iCOGS	734	957
GC-HBOC	Germany	BRIDGES, OncoArray, iCOGS	1086	1732
GENICA	Germany	BRIDGES, OncoArray, iCOGS	1005	963
GENSCOT	UK	BRIDGES	427	761
GESBC	Germany	BRIDGES, OncoArray	588	1006
HABCS	Germany	BRIDGES, OncoArray	1017	900
HMBCS	Belarus	BRIDGES, OncoArray, iCOGS	867	506
HUBCS	Russia	BRIDGES, OncoArray	259	213
KARBAC	Sweden	BRIDGES, OncoArray, iCOGS	789	662
KARMA	Sweden	BRIDGES, OncoArray	4001	14942
KBCP	Finland	BRIDGES, OncoArray, iCOGS	587	436
KCONFAB/AOCS	Australia	BRIDGES, iCOGS	288	905
MARIE	Germany	BRIDGES, OncoArray, iCOGS	2551	2068
MASTOS	Cyprus	BRIDGES	709	1094
MBCSG	Italy	BRIDGES, OncoArray, iCOGS	214	766
MCCS	Australia	BRIDGES, OncoArray, iCOGS	1252	1209
NBCS	Norway	BRIDGES, OncoArray, iCOGS	2606	789
OFBCR	Canada	BRIDGES, OncoArray, iCOGS	1873	1140
ORIGO	Netherlands	BRIDGES, OncoArray, iCOGS	1384	1614
PBCS	Poland	BRIDGES, OncoArray, iCOGS	1878	2082
PKARMA	Sweden	BRIDGES, OncoArray, iCOGS	5606	5459
PLCO	USA	BRIDGES, OncoArray	2408	2605
PROCAS	UK	BRIDGES, OncoArray	477	1651
RBCS	Netherlands	BRIDGES, OncoArray, iCOGS	127	933
SASBAC	Sweden	BRIDGES, iCOGS	1170	1381
SEARCH	UK	BRIDGES, OncoArray, iCOGS	14135	9643
SKKDKFZS	Germany	BRIDGES, OncoArray, iCOGS	1233	29
SZBCS	Poland	BRIDGES, OncoArray, iCOGS	705	493
UBCS	USA	BRIDGES, OncoArray	833	306
2SISTER	USA	OncoArray	1070	0
ABCFS	Australia	OncoArray, iCOGS	1440	738
ABCTB	Australia	OncoArray	926	375
AHS	USA	OncoArray	514	1137
BBCS	UK	OncoArray, iCOGS	1661	1839
BCEES	Australia	OncoArray	783	835
BCFR-NY	USA	OncoArray	453	27
BCFR-PA	USA	OncoArray	73	0
BCFR-UTAH	USA	OncoArray	101	0
BCINIS	ISRAEL	OncoArray	1437	724

CBCS	CANADA	OncoArray	676	817
CPSII	USA	OncoArray, iCOGS	3165	3324
CTS	USA	OncoArray, iCOGS	1175	648
DIETCOMPLYF	UK	OncoArray	711	0
EPIC	France, Germany, Italy, Netherlands, Spain, UK	OncoArray	3668	3461
ESTHER	Germany	OncoArray, iCOGS	479	507
FHRISK	UK	OncoArray	20	38
GEPARSIXTO	Germany	OncoArray	387	0
HCSC	Spain	OncoArray	426	0
LMBC	Belgium	OncoArray, iCOGS	3474	1823
MABCS	Republic of North Macedonia	OncoArray	82	92
MCBCS	USA	OncoArray, iCOGS	3083	2102
MEC	USA	OncoArray, iCOGS	1123	1123
MISS	Sweden	OncoArray	701	1545
MMHS	USA	OncoArray	374	1635
MSKCC	USA	OncoArray	138	0
MTLGEBCS	Canada	OncoArray, iCOGS	533	465
NBHS	USA	OncoArray, iCOGS	684	731
NC-BCFR	USA	OncoArray	774	151
NCBCS	USA	OncoArray	2389	1006
NHS	USA	OncoArray	1436	1804
NHS2	USA	OncoArray	1521	1905
POSH	UK	OncoArray	1088	0
PREFACE	Germany	OncoArray	2954	0
SBCS	UK	OncoArray	940	848
SISTER	USA	OncoArray	2001	1557
SMC	Sweden	OncoArray	1509	704
SUCCESSB	Germany	OncoArray	440	0
SUCCESSC	Germany	OncoArray	2836	0
TNBCC	Germany, USA	OncoArray, iCOGS	869	424
UCIBCS	USA	OncoArray	501	258
UKBGS	UK	OncoArray, iCOGS	1661	1031
UKOPS	UK	OncoArray	0	974
USRT	USA	OncoArray	1692	1699
WHI	USA	OncoArray	5095	4455
ABCS-F	Netherlands	iCOGS	968	0
GLACIER	UK	iCOGS	2322	0
ICICLE	UK	iCOGS	2901	1
OBCS	Finland	iCOGS	506	414

[1] One study contributing to previous analyses (HEBCS) was subsequently withdrawn from the BCAC

[2]Includes invasive breast cancer and carcinoma in-situ of the breast

Supplemental table 2: Estimated odds ratios for the association of *ATM* c.7271T>G with invasive breast cancer (BC) and carcinoma in-situ of the breast (CIS), by panel, in the BCAC dataset

**Outcome, dataset**	**Case carriers**	**Case non-carriers**	**Control carriers**	**Control non-carriers**	**Model adjusted by country and panel**			**Model adjusted by country, panel, and principal components**		
					**OR**	**95% CI**	***P*-value**	**OR**	**95% CI**	***P*-value**
BC combined	43	114736	10	115482	4.11	2.05 to 8.26	6.9E-05	4.13	2.05 to 8.32	7.3E-05
BC BRIDGES	11	37988	6	42935	1.86	0.66 to 5.20	0.24	NA	NA	NA
BC OncoArray	27	53458	3	47208	7.15	2.14 to 23.9	0.001	7.10	2.13 to 23.7	0.0014
BC iCOGS	5	23290	1	25339	6.57	0.76 to 57.1	0.09	6.87	0.79 to 59.5	0.080
CIS combined	7	11638	10	115482	9.30	2.69 to 32.2	4.3E-04	9.85	2.84 to 34.1	3.1E-04
CIS BRIDGES	1	2046	6	42935	5.46	0.34-87.8	0.23	NA	NA	NA
CIS OncoArray	5	5189	3	47208	11.6	2.70 to 49.8	9.7E-04	11.7	2.72 to 50.2	9.4E-04
CIS iCOGS	1	4403	1	25339	1.72	0.11 to 27.5	0.70	1.88	0.12 to 30.5	0.66

Supplemental table 3: Odds ratios, hazard ratio, and pooled estimates for the association of c.7271T>G in *ATM* with cancer by type in UKB whole-exome sequence data

	Retrospective Analysis				Prospective study				Pooled analysis	
**Cancer**	**Carrier count**	**Non-carriers**	**OR (95% CI)**	***P*-value**	**Carrier count**	**Non-carriers**	**HR (95% CI)**	***P*-value**	**Relative Risks (95% CI)**	***P*-value**
Breast	3	6299	2.96 (0.90 to 9.69)	0.074	5	6196	5.79 (2.41 to 13.9)	8.7E-05	4.57 (2.25 to 9.30)	2.7E-05
Prostate	3	2559	6.69 (2.04 to 22.0)	1.7E-03	4	8161	3.89 (1.46 to 10.4)	6.6E-03	4.84 (2.27 to 10.33)	4.5E-05
Carcinoma in-situ of the breast	1	954	6.63 (0.90 to 48.7)	0.063	2	1109	14.28 (3.56 to 57.3)	1.8E-04	11.12 (3.55 to 34.84)	3.6E-05
Any cancer	7	18830	2.20 (1.00 to 4.84)	0.049	22	37779	4.19 (2.76 to 6.37)	1.8E-11	3.64 (2.53 to 5.24)	3.7E-12
Any cancer in females	4	11831	2.13 (0.75 to 6.05)	0.16	11	17701	4.49 (2.49 to 8.12)	6.4E-07	3.74 (2.24 to 6.23)	4.5E-07
Any cancer in females minus breast	1	5559	1.07 (0.15 to 7.85)	0.95	6	11551	3.76 (1.69 to 8.37)	1.2E-03	3.15 (1.49 to 6.63)	0.0026
Any cancer in males	3	6999	2.32 (0.71 to 7.62)	0.17	11	20078	4.15 (2.30 to 7.50)	2.4E-06	3.70 (2.18 to 6.26)	1.2E-06
Any cancer in males minus prostate	0	4440			7	11917	4.31 (2.05 to 9.04)	1.1E-04	2.79 (1.33 to 5.85)	0.0066

Supplemental table 4: Other cancers identified in *ATM* c.7271T>G carriers in UKB data

Cancer	Carrier count	Non-carriers
Other and unspecified parts of tongue	1	111
Stomach	1	481
Colon	1	2761
Rectum	1	1164
Other and unspecified parts of biliary tract	1	132
Pancreas	2	882
Bladder	1	786
Follicular non-Hodgkin's lymphoma	1	327
Diffuse non-Hodgkin's lymphoma	1	795
Lymphoid leukaemia	1	511

Supplemental figures

Supplemental figure 1a: Cluster plot of normalised *ATM* c.7271T>G genotype intensities in the Oncoarray dataset

Supplemental figure 1b: Cluster plot of normalised *ATM* c.7271T>G genotype intensities in the iCOGS dataset

Supplemental figure 1a. Cluster plot of normalised *ATM* c.7271T>G genotype intensities in the Oncoarray dataset

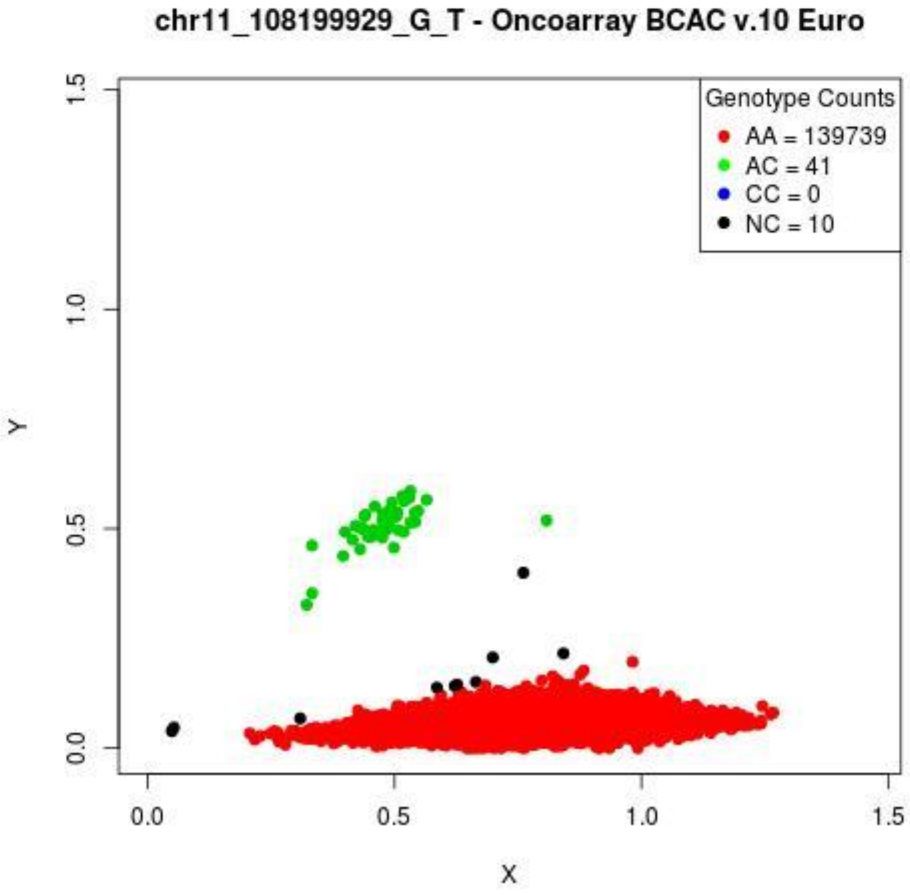

Supplemental figure 1b. Cluster plot of normalised *ATM* c.7271T>G genotype intensities in the iCOGS dataset

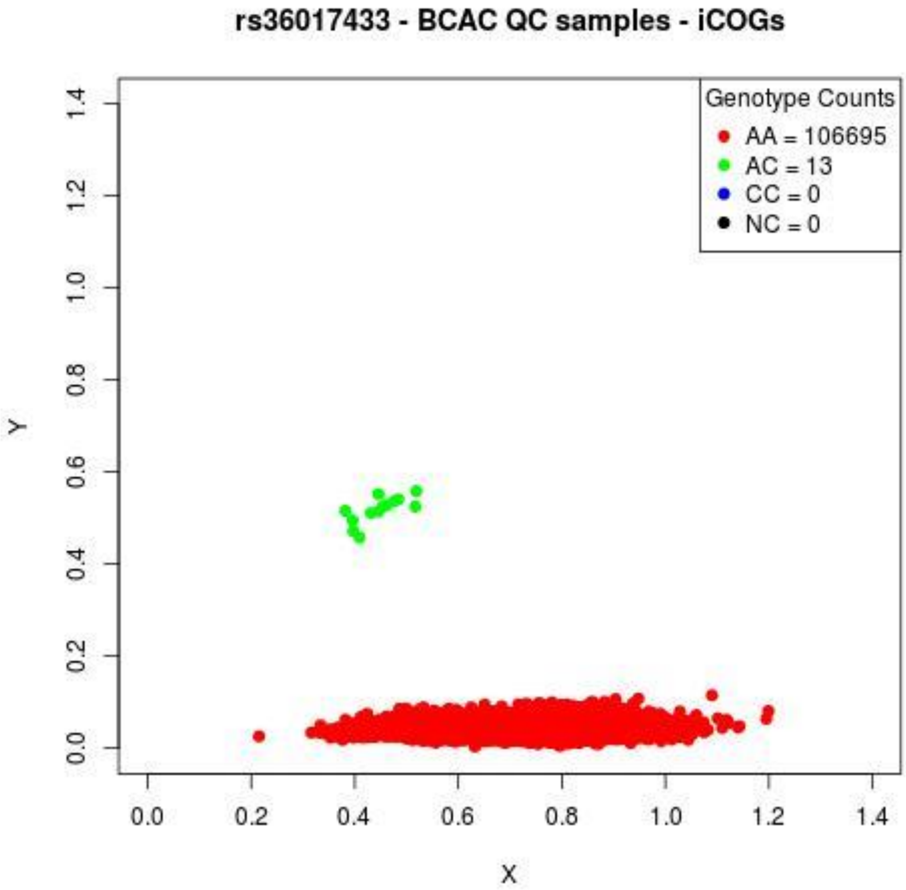

Supplement: online supplemental file 1 [file jmg-62-9-s001.pdf]
